# Evaluation of a Proteinase K-Based Extraction Method to Detect Hepatitis A Virus, Hepatitis E Virus and Norovirus in Artificially Contaminated Dairy Products

**DOI:** 10.3390/foods12071489

**Published:** 2023-04-01

**Authors:** Catherine Hennechart-Collette, Lisa Fourniol, Audrey Fraisse, Sandra Martin-Latil, Sylvie Perelle

**Affiliations:** Laboratory for Food Safety, Université Paris-Est, Anses, F-94700 Maisons-Alfort, France

**Keywords:** milk products, human norovirus, hepatitis virus (A, E), proteinase K sample treatment, evaluation, RT-qPCR, detection, process control

## Abstract

Human norovirus and hepatitis viruses (hepatitis A (HAV) and hepatitis E (HEV)) are leading causes of foodborne disease worldwide. Among the various food products, different types of dairy products can be implicated in viral foodborne outbreaks and contamination can occur at different stages, such as preparation, contact with contaminated equipment or via other foods. The aim of this study was to characterise a proteinase K method adapted from the ISO 15216 method for the detection of HAV, HEV and norovirus in artificially contaminated dairy products, based on the recent international standard of ISO 16140-4. Results showed that the recovery yields obtained from pure RNA in dairy products ranged from 5.76% to 76.40% for HAV, from 35.09% to 100.00% for HEV, from 25.09% to 100.00% for norovirus GI and from 47.83% to 100.00% for norovirus GII. The process control MNV-1 was detected in all RNA extracts, with recovery yields between 36.83% and 100.00%. The limit of detection (LOD) of the method was between 184 and 642 genome copies/mL (or/g) for the LOD_50_ and 802 and 2800 genome copies/mL or/g for the LOD_95_ according to the virus analysed. This method proved to be suitable for detecting viruses in dairy products for routine diagnostic needs.

## 1. Introduction

A wide range of viruses is implicated in foodborne outbreaks. In 2019, viruses were responsible for 11% of foodborne outbreaks in Europe [1]. Among the enteric viruses, hepatitis viruses (hepatitis A (HAV) and hepatitis E (HEV)) and human norovirus are leading causes of foodborne disease. Norovirus is the second-most frequently reported agent in foodborne outbreaks, and hepatitis A was involved in 22 outbreaks in Europe in 2019 [1]. Similarly, between 2009 and 2018 in the United States, norovirus was responsible for 47% of foodborne illnesses [2]. Enteric viruses are mainly transmitted via the faecal–oral and vomit–oral routes, including direct person-to-person contact, the consumption of contaminated food or water, contact with contaminated environmental surfaces [3,4,5,6,7,8] and, for HEV, direct contact with infected animals [9].

A wide variety of foodstuffs, such as molluscs and fresh fruits and vegetables, are frequently involved in foodborne disease outbreaks [10,11,12,13,14]. In addition to these high-risk foodstuffs, dairy products can be associated with enteric virus outbreaks [15,16,17,18,19]. The contamination of these dairy products mainly occurs via milk used to prepare products, during the dairy products manufacturing stage, in restaurants during food preparation by infected workers, through contact with contaminated equipment or via other foods, such as dairy products with added fruit.

A method for detecting viruses in dairy products is needed to ensure the safety of these products. The ISO 15216 procedures involve standard molecular methods to detect noroviruses and HAV in fruits, vegetables, water and bivalve molluscs [20,21], but do not offer a standardised method for the detection of viruses in dairy products, nor for the detection of HEV in foodstuffs. Recently, methods have been developed to recover norovirus and hepatitis viruses from milk [22,23,24,25], cheese [26,27] or cottage cheese [24], and a method using proteinase K has been applied to detect noroviruses in dairy products in virological outbreak investigations [15].

The purpose of this study was to evaluate a proteinase K-based extraction method to detect hepatitis A virus, hepatitis E virus and norovirus in artificially contaminated dairy products. Due to the low level of viral contamination in the food and the presence of fats, casein, whey proteins and lactose in milk products, a comprehensive set of controls must be used [28,29,30]. The ISO 15216 method includes the use of a process control virus and external amplification controls (EACs), such as external control RNA, to assess any inhibition of amplification [20,21]. The ISO 16140 procedure establishes the general principle, as well as the technical protocol, for the validation of alternative methods in the field of microbiological analysis of food. The recent international standard ISO 16140-4:2018 describes experimental designs to test the effect of matrices, virus inoculum levels and interaction between various factors, and also reflects the variation within a single laboratory under routine conditions. The proteinase K-based extraction method for the detection of HAV, HEV and noroviruses in artificially contaminated dairy was characterised according to the recently published ISO 16140-4 method (Microbiology of the food chain—Method validation—Part 4: Protocol for single-laboratory method validation) [31].

## 2. Materials and Methods

### 2.1. Viruses

The HAV, HEV, norovirus and murine norovirus (MNV-1) stocks were prepared and titrated as described in [32]. The genomic titters of viruses were determined using an RT-qPCR standard curve obtained with the 10-fold diluted in vitro HAV, HEV, norovirus GI, norovirus GII and MNV-1 RNA transcripts. The RNA transcripts were quantified by measuring absorbance at 260/280 nm with a spectrophotometer.

HAV strain HM175/18f (clone B (VR-1402)) and a clarified HEV (genotype 3e) suspension from faecal samples of infected swine had, respectively, a titre of 1.10 × 10^9^ and 1.00 × 10^7^ genome copies/mL. The clarified faecal suspension stocks from humans infected with norovirus GI.3 (E16518) or norovirus GII.4 (E16461) had titres of 2.90 × 10^6^ and 9.90 × 10^6^ genome copies/mL, respectively. The stock of MNV-1 had a titre of 8.98 × 10^11^ genome copies/mL.

### 2.2. Inoculation of Dairy Products

Sixteen dairy product samples (Table 1) purchased from a supermarket were artificially contaminated with 100 μL of clarified virus suspensions at four concentrations of HAV, HEV, norovirus GI and norovirus GII. The ISO 16140-4:2018 experimental design was applied as described in [32,33] (Table 2).

Viral stocks serially diluted 10-fold in DEPC-treated ultrapure water were used for HAV, HEV and norovirus inocula. The inoculum levels ranged per sample, from 1.10 × 10^2^ to 1.10 × 10^5^ genome copies for HAV, 1.00 × 10^2^ to 1.00 × 10^5^ genome copies for HEV, 2.90 × 10^2^ to 2.90 × 10^5^ genome copies for norovirus GI and 9.90 × 10^1^ to 9.90 × 10^4^ genome copies for norovirus GII.

Samples were spiked with one virus at a time and were co-inoculated with MNV-1, which was used as the process control virus (8.98 × 10^8^ genome copies).

### 2.3. Viral Detection

**Figure 1:**
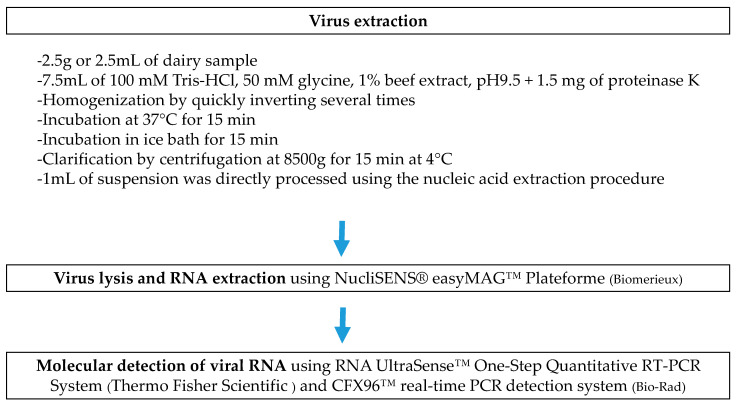
Flow diagram of the proteinase K method for the recovery and the detection of viruses in dairy samples.

The proteinase K-based extraction method used to recover virus particles from dairy products was previously adapted from the ISO 15216 shellfish method [20,21], as described in [24]. Figure 1 describes the method used for the recovery and the detection of viruses in dairy samples.

Viral RNA extraction, the set of primers and probes used for the molecular detection and one-step quantitative real-time RT-PCR amplifications were previously described by [32,33].

The percentage of virus recovery was calculated following this formula: quantity of virus recovered after spiking experiments × (volume of elution buffer)/quantity of viral inoculum × 100.

RNA transcripts for each viral target were used as an EAC to monitor real-time RT-PCR inhibition in dairy samples, as described in ISO 15216. The inhibition rates in extracted RNA were calculated using the following formula: 100 − (quantity of external control RNA detected in sample/quantity of external control RNA detected in ultrapure water × 100).

### 2.4. Limits of Detection Values

The limits of detection (LOD_50_) and (LOD_95_), corresponding, respectively, to 50% and 95% of the probability of the viral detection in samples were calculated as described in Wilrich et al. [34] by using the POD LOD calculation software (version 9, dated 23 September 2017) (www.wiwiss.fu-berlin.de/fachbereich/vwl/iso/ehemalige/wilrich/index.html) (accessed on 23 september 2017).

### 2.5. Statistical Analysis

Statgraphics Centurion XVII software (Version 17.1.04) was applied for statistical analyses.

To test (1) the effect of the viruses (HAV, HEV, norovirus and MNV-1) on virus recovery rates, and (2) the dilution of RNA extracts (pure vs. 10-fold diluted) on viral recovery rates, a one-way analysis of variance (ANOVA) was first used. The result of the ANOVA was a *p*-value associated with the hypothesis that the mean recovery rates of all groups were the same. Because the extraction yields were statistically different (ANOVA, *p* < 0.01), a multiple comparison procedure (Fisher’s least-significant differences (LSD)) was applied to determine which viruses provided the highest extraction yields. Two-way ANOVA was then applied to study the influence of additional factors on extraction yields of HAV, HEV and norovirus regarding (1) the experiment set (R1 to R4) and (2) the viral concentration.

## 3. Results

The proteinase K-based extraction method was characterised on sixteen different types of artificially contaminated dairy samples. The influence of experimental factors on the extraction yields of pathogenic viruses was assessed.

### 3.1. Mean Recoveries of HAV, HEV and Norovirus and Limits of Detection

The mean recovery yields obtained for HAV, HEV, norovirus GI and norovirus GII in dairy products according to the inoculum level and the experiment set (R1 to R4) are reported in Table 3. The average recovery rates with pure RNA extracts ranged from 5.76% to 76.40% for HAV, from 35.09% to 100.00% for HEV, from 25.09% to 100.00% for norovirus GI and from 47.83% to 100.00% for norovirus GII.

The ratio between mean extraction yields obtained with undiluted RNA extracts and those obtained with 10-fold diluted extracts (F factor) ranged from 0.1 to 1.63.

The LOD values were calculated for HAV, HEV, norovirus GI and norovirus GII for all 16 samples and are presented in Table 4. The LOD_50_ were between 184 and 642 genome copies/mL or /g and the LOD_95_ were between 802 and 2800 genome copies/mL or /g.

### 3.2. MNV-1 and EAC Results

According to the ISO 15216 procedure, the recovery rate of the process control should be higher than 1% and the rates of inhibition in RNA extracted from food samples should be lower than 75%. Data of MNV-1 are presented in Table 3. MNV-1 was detected in all analysed RNA extracts and was recovered with an efficiency of between 36.83% and 100%.

The implementation of the EAC corresponding to all viral targets was used to examine quantitative real-time RT-PCR inhibition. Table 5 shows the mean inhibition rates of quantitative real-time RT-PCR reaction for each of the 16 food samples. For most of the foodstuffs analysed, the inhibition rate was less than 20%, except for the vanilla dessert cream (46.7%).

### 3.3. Experimental Factors on Virus Extraction Yield

**Figure 2:**
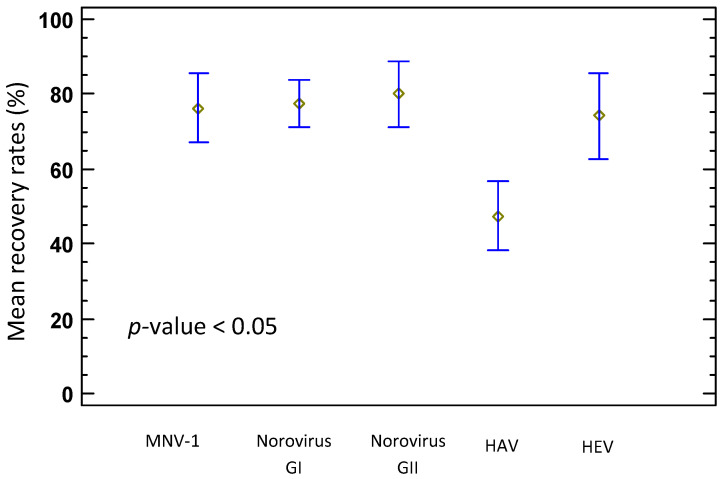
Comparison of mean recovery rates of viruses from spiked dairy products across all experiments.

For the characterisation of the method, the influence of experimental factors (viruses, RNA extract dilution, the experiment sets and the virus inoculum levels) on extraction yields of pathogenic viruses was assessed. The statistical results showed that the virus recovery rates from dairy products varied with the virus inoculated (ANOVA; *p*-value < 0.0001), and the multiple comparison tests showed that the recovery rates of HAV were significantly lower (Figure 2).

No significant impact of RNA extract dilution on recovery rates was observed (ANOVA; *p*-value = 0.3040).

The statistical analysis showed that the differences among the experiment sets R1 to R4 were not significant for the extraction yields of HAV (ANOVA; *p*-value = 0.1647), norovirus GI (ANOVA; *p*-value = 0.6256) or norovirus GII (ANOVA; *p*-value = 0.4492), but were significant for the extraction yield of HEV (ANOVA; *p*-value = 0.0003). More specifically, the multiple comparison tests showed that R3 and R4 were significantly different to the R1 and R2 sets.

The statistical analysis showed that the recovery rates of HAV and norovirus GII were not statistically different among the virus inoculum levels (ANOVA; *p*-values = 0.0561 and 0.1565 for HAV and norovirus GII, respectively), but were significant for HEV (ANOVA; *p*-value = 0.0002) and norovirus GI (ANOVA; *p*-value = 0.0000). More specifically, the multiple comparison tests showed that inoculation with 1.00 × 10^5^ genome copies of HEV was significantly different to the other HEV inoculum levels, and 2.90 × 10^5^ genome copies and 2.90 × 10^3^ genome copies of norovirus GI were significantly different to the other norovirus GI inoculum levels.

## 4. Discussion

Food poisoning outbreaks may be associated with a wide variety of foods, including dairy products. Dairy products are consumed in different forms, such as milk, yoghurt or cheese, and can be associated with viral foodborne outbreaks. Contamination can occur at different stages of production, processing and storage [35]. In the United States, outbreaks due to cheese made from pasteurised milk are most often caused by norovirus contamination, arising during cheese making, through contact with contaminated equipment or via infected food workers [36]. In France, during virological investigations of outbreaks occurring in catering settings, noroviruses have been detected in various dairy products such as pastry creams or smoothies [15]. In addition to the risk associated with food handlers, fruit added to dairy products may lead to food contamination with viruses. Indeed, fruits belong to the high-risk foods group for virus contamination and, recently, European countries have faced an increase in norovirus and HAV outbreaks from the consumption of raspberries, strawberries and other types of berries [37,38,39,40,41].

Although pasteurisation can eliminate pathogenic microorganisms from milk, the consumption of unpasteurised and contaminated milk represents a viral risk for consumers. The European Food Safety Authority (EFSA) has suggested that the consumption of unpasteurised milk products represents a source of HEV infection for consumers [42]. Prevalent studies have shown that, in contrast to Germany and Belgium, where no HEV RNA has been detected in cow’s milk samples [43,44], the detection of HEV RNA in cow’s, sheep’s, goat’s or camel’s milk in other countries suggests the possible transmission of HEV through the ingestion of contaminated milk [23,45,46,47,48,49]. In these cases, poor hygiene and the use of contaminated water may also be involved. Milk contamination by HEV may also occur via pastures contaminated by wild animals and frequented by small domestic ruminants [23]. One study has reported that HEV isolated from cow’s milk belonged to the same subtype as that found in pigs and humans in the same region [50].

In our study, the method for HEV detection in different milk samples was evaluated and should be used to assess potential HEV health risks in these products. However, the possible HEV excretion in milk of a wide variety of infected animals (cows, goats, donkeys, buffaloes, sheep and camels) [51] highlights the need to evaluate the stability of HEV in these milks, and to evaluate the infectious risk of HEV in dairy products. The proteinase K-based extraction method evaluated in this study to detect HAV, HEV and norovirus in artificially contaminated dairy products cannot be used to determine the infectivity of the virus by using molecular methods to detect viral genomes from intact viral particles. To our knowledge, currently there is no RT-qPCR integrity method developed for the detection of HEV in milk products and no infectious titration method for HEV has been developed. On the other hand, the detection of viruses in milk products can be difficult because dairy products are rich in casein, whey proteins, lactose and fat. The presence of these components in samples could affect nucleic acid isolation, since it is known that the RNA molecule is overly sensitive to lyse and temperature variation. Proteinase K, which is used in genetic material extraction protocols, digests proteins and thus eliminates contamination from nucleic acids preparations. It further inactivates nucleases that could degrade DNA/RNA during purification.

In this study, according to the ISO procedure, process control virus and EAC were both included in our characterisation of the method. The MNV-1 used as a process control was detected in all analysed RNA extracts and was recovered with an efficiency between 36.83% and 100%. The MNV-1 recovery rate was higher than 1% in all analysed samples and was in the same range as HAV, HEV and norovirus recovery rates. A previous study used Mengovirus as a process control virus for a comparison of extraction methods to detect Tick-borne encephalitis virus in milk and cream cheese and showed that the Mengovirus recovery rate was between 1.2% and 18.8% depending on the method used [52]. Another study using the proteinase K method to detect noroviruses in dairy products has evaluated the use of MNV-1 and Mengovirus as process control viruses and showed that their recovery efficiencies were, respectively, 60.59% and 79.23% [24]. The inhibition rate was less than 20% for most of the foodstuffs analysed. Unlike other matrices, such as red fruits or vegetables, the dairy products analysed in this study showed little PCR reaction inhibition [53]. However, to our knowledge, there are no data involving both of these controls in the characterisation of a method for detecting enteric viruses from various milk products including milk, cheese, yoghurt and custard.

Here, we applied an adapted experimental design described in ISO 16140-4:2018 to characterise a method for the detection of viruses in dairy products. In addition to the factors studied in the ISO 16140 standard, part 4 describes the calculation of repeatability and reproducibility. The experimental design for this procedure is sufficient for its characterisation without a reference method. The ISO 16140-4 design considers the information on the sources of variation within a laboratory or across relevant matrices. In our study, 16 different dairy product samples that could potentially be involved in viral outbreaks, including milk, cheese, yoghurt and cream, were tested. A wide variety of food samples is essential to assess the performance of a method. To our knowledge, this is the first time that the ISO 16140-4 method has been used to characterise a method to detect HAV, HEV and noroviruses from milk, cheese, yoghurt or cream simultaneously.

In this study, the laboratory technician factor was not taken into account. Other studies have shown that the technician does not influence virus recoveries from drinking water or from multicomponent foodstuffs [32,33]. Here, virus recovery rates varied with the virus, corroborating data reported in a previous study [33]. The differential behaviour of the spiked viruses depends on several factors, such as food type, method and the virus itself [54,55,56].

The HAV and norovirus recovery rates obtained in this study ranged from 5.76% to 76.40% and from 25.09% to 100.00%, respectively. These data were in agreement with other reported data for HAV in milk with strawberry, yoghurt with soft fruit and ice cream with soft fruit [22], and for norovirus in milk, cottage cheese and cheese [24,27]. The HEV recovery rates obtained in this study, ranging from 35.09% to 100.00%, were also in agreement with other reported HEV data in milk [57].

The extraction yield obtained for HAV varied significantly according to the experiment set, and the recovery rates of HEV and norovirus GII varied significantly according to the inoculum levels. This variability could be attributed to the variety of milk product components such as fats, sugars or caseins [26,58,59,60].

In the present study, for all dairy products analysed, LOD_95_ values were between 802 and 2800 genome copies/mL or /g, regardless of the virus analysed. Using the same proteinase K method, another study reported detection limits of norovirus in milk and cottage cheese that were in agreement with our data and were, respectively, between 10^3^ and 10^5^ genome copies per g or mL, according to genogroup [24]. The LOD of HEV in milk with other extraction methods fell between 3 × 10^2^ and 6 × 10^3^ IU/mL [25,57], and for norovirus in cheese the LOD was 2.7 × 10^3^ genome copies/g [26].

In conclusion, our results showed that the proteinase K-based extraction method is effective at detecting enteric viruses in dairy product matrices. Our study underscores the importance of developing, standardising and validating a laboratory method for detecting viruses in other food matrices, or for detecting viruses other than those described in the ISO 15216 procedure. The described method can be applied to dairy products for routine diagnostic needs to determine the importance of dairy products in viral foodborne outbreaks and to assess potential health risks.

## Figures and Tables

**Table 1:** Selected dairy products included in the study.

Forms of Dairy Products	Types of Dairy Products
Cheese	Goat’s cheese
	Sheep’s cheese
	Camembert Raclette
	Emmental
	Cheddar Mozzarella Feta
Milk	Pasteurised whole cow’s milk
	Semi-skimmed cow’s milk Pasteurised goat’s milk
Yoghurt and dessert cream	Vanilla dessert cream Raspberry pulp stirred yoghurt
	Raspberry-flavoured yoghurt drink Raspberry-flavoured yoghurt Cottage cheese with fruit

**Table 2:** Experimental design for detection of hepatitis virus A (HAV), hepatitis virus E (HEV) and norovirus (genogroups GI and GII) in dairy products adapted from the ISO 16140-4:2018 procedure.

	Experiment Set				
Virus inoculum levels		R1	R2	R3	R4
	High	Pasteurised whole cow’s milk	Semi-skimmed cow’s milk	Cheddar cheese	Raspberry-flavoured yoghurt drink
	Medium	Goat’s cheese	Camembert	Pasteurised goat’s milk	Emmental cheese
	Low	Vanilla dessert cream	Raspberry yoghurt (blended)	Mozzarella cheese	Raspberry-flavoured yoghurt
	Very low	Sheep’s cheese	Feta	Cottage cheese with fruit	Raclette cheese

**Table 3:** Virus recovery (%±SD) calculated for four inoculum levels of HAV, HEV, norovirus GI or norovirus GII in the presence of MNV-1. The ratio between mean values of extraction yields obtained with undiluted RNA extracts and those obtained with 10-fold diluted RNA extracts (F) were calculated to determine whether the dilution of RNA extracts enhanced mean extraction yields.

Virus	Inoculum Level (Number of Genome Copies)	RNA Extract	Experiment Set R1 (%±SD)	(F)	Experiment Set R2 (%±SD)	(F)	Experiment Set R3 (%±SD)	(F)	Experiment Set R4 (%±SD)	(F)
HAV	1.1 × 10^5^	Pure	36.95 ± 0.40 (2/2)	1.63	33.09 ± 0.70 (2/2)	1.35	70.06 ± 4.57 (2/2)	1.13	74.71 ± 4.88 (2/2)	0.93
		10-fold diluted	60.27 ± 4.52 (2/2)		44.80 ± 20.87 (2/2)		79.02 ± 12.39 (2/2)		69.31 ± 9.60 (2/2)	
	1.1 × 10^4^	Pure	59.23 ± 4.97 (2/2)	0.33	40.41 ± 1.20 (2/2)	0.70	66.72 ± 7.64 (2/2)	0.16	76.40 ± 8.58 (2/2)	0.46
		10-fold diluted	19.38 ± 23.92 (2/2)		28.31 ± 36.00 (2/2)		10.45 (1/2)		35.43 (1/2)	
	1.1 × 10^3^	Pure	11.26 (1/2)	-	5.76 ± 0.33 (2/2)	-	10.77 ± 13.52 (2/2)		not detected	
		10-fold diluted	not detected		100.00 (1/2)		not detected		not detected	
	1.1 × 10^2^	Pure	not detected	-	not detected		not detected		not detected	
		10-fold diluted	not detected		not detected		not detected		not detected	
MNV-1	MNV-1	Pure	90.52 ± 0.75 (4/4)		36.83 ± 0.43 (4/4)		81.68 ± 0.20 (4/4)		100.00 (4/4)	
	Nb of MNV-1 samples > 1%	16/16								
HEV	1.0 × 10^5^	Pure	48.27 ± 11.94 (2/2)	0.90	53.16 ± 3.72 (2/2)	0.80	100.00 (2/2)	1	56.46 ± 2.93 (2/2)	0.1
		10-fold diluted	43.61 ± 1.63 (2/2)		42.32 ± 15.30 (2/2)		100.00 (2/2)		5.63 ± 0.39 (2/2)	
	1.0 × 10^4^	Pure	100.00 (2/2)	1	100.00 (2/2)	1	100.00 (2/2)	1	100.00 (2/2)	-
		10-fold diluted	100.00 (2/2)		100.00 (1/2)		100.00 (1/2)		not detected	
	1.0 × 10^3^	Pure	not detected	-	not detected	-	not detected	-	35.09 (1/2)	-
		10-fold diluted	not detected		100.00 (1/2)		not detected		not detected	
	1.0 × 10^2^	Pure	100.00 (1/2)	-	100.00 (1/2)	-	not detected	-	not detected	-
		10-fold diluted	not detected		not detected		not detected		not detected	
MNV-1	MNV-1	Pure	72.27 ± 0.50 (4/4)		71.15 ± 0.49 (4/4)		100.00 (4/4)		100.00 (4/4)	
	Nb of MNV-1 samples > 1%	16/16								
Norovirus GI	2.90 × ^105^	Pure	65.04 ± 7.72 (2/2)	0.75	64.09 ± 1.46 (2/2)	1.14	80.87 ± 13.86 (2/2)	0.80	58.91 ± 6.14 (2/2)	1.01
		10-fold diluted	48.75 ± 30.10 (2/2)		73.22 ± 5.67 (2/2)		65.22 ± 5.63 (2/2)		59.72 ± 7.86 (2/2)	
	2.90 × 10^4^	Pure	88.58 ± 3.18 (2/2)	1.13	71.33 ± 5.78 (2/2)	1.40	68.72 ± 1.94 (2/2)	1.31	100.00 (2/2)	1
		10-fold diluted	100.00 (2/2)		99.83 ± 12.87 (2/2)		90.05 ± 7.02 (2/2)		100.00 (2/2))	
	2.90 × 10^3^	Pure	25.09 (1/2)	-	65.12 ± 1.54 (2/2)	1.08	100.00 (2/2)	1	100.00 (2/2)	0.19
		10-fold diluted	not detected		70.78 ± 7.43 (2/2)		100.00 (2/2)		19.48 ± 19.15 (2/2)	
	2.90 × 10^2^	Pure	100.00 (2/2)	1	100.00 (2/2)	-	not detected	-	100.00 (2/2)	-
		10-fold diluted	100.00 (1/2)		not detected		not detected		not detected	
MNV-1	MNV-1	Pure	74.23 ± 0.78 (4/4)		100.00 (4/4)		100.00 (4/4)		100.00 (4/4)	
	Nb of MNV-1 samples > 1%	16/16								
Norovirus GII	9.9 × 10^4^	Pure	64.50 ± 6.43 (2/2)	1.40	84.20 ± 6.43 (2/2)	0.47	100.00 (2/2)	0.89	92.94 ± 7.76 (2/2)	1.07
		10-fold diluted	90.87 ± 13.51 (2/2)		39.77 ± 15.43 (2/2)		89.86 ± 24.25 (2/2)		100.00 (2/2)	
	9.9 × 10^3^	Pure	100.00 (2/2)	1	100.00 (2/2)	1	78.76 ± 14.93 (2/2)	0.72	59.23 ± 60.40 (2/2)	-
		10-fold diluted	100.00 (2/2)		100.00 (1/2)		57.20 (1/2)		not detected	
	9.9 × 10^2^	Pure	not detected	-	47.83 ± 62.15 (2/2)	-	49.13 (1/2)	-	100.00 (1/2)	-
		10-fold diluted	100.00 (2/2)		not detected		not detected		not detected	
	9.9 × 10^1^	Pure	100.00 (1/2)	-	100.00 (1/2)	-	100.00 (1/2)	-	not detected	-
		10-fold diluted	not detected		not detected		not detected		not detected	
MNV-1	MNV-1	Pure	74.27 ± 0.59 (4/4)		91.34 ± 0.59 (4/4)		98.85 ± 0.70 (4/4)		100.00 (4/4)	
	Nb of MNV-1 samples > 1%	16/16								

(Ct): The number of positive cycle threshold (Ct) determinations is mentioned for HAV, HEV, norovirus GI and GII. RNA extracts were tested twice, resulting in two Ct values for each sample. Negative samples were not considered while calculating the recoveries. Extraction yields greater than 100% were rounded down to 100%.

**Table 4:** LOD_50_ and LOD_95_ for HEV, HAV and norovirus across all experiment sets.

	Target Virus			
Per g or Per mL of Dairy Product	HEV	HAV	Norovirus GI	Norovirus GII
LOD_50_	6.42 × 10^2^	3.62 × 10^2^	1.84 × 10^2^	2.48 × 10^2^
LOD_95_	2.80 × 10^3^	1.56 × 10^3^	8.02 × 10^2^	1.07 × 10^3^

**Table 5:** Mean percentage of RT-qPCR inhibition rates for each RNA sample.

Sample	RT-qPCR Inhibition (%±SD)
Pasteurised whole cow’s milk	0.99 ± 1.86 (N = 4)
Semi-skimmed cow’s milk	7.96 ± 10.45 (N = 4)
Pasteurised goat’s milk	0.00 (N = 4)
Raspberry-flavoured drinkable yoghurt	0.00 (N = 4)
Vanilla dessert cream	46.71 ± 23.98 (N = 4)
Raspberry pulped stirred yoghurt	16.08 ± 23.73 (N = 4)
Cottage cheese with fruits	13.77 ± 12.87 (N = 4)
Raspberry-flavoured yoghurt	0.00 (N = 4)
Goat’s cheese (cheese)	9.41 ± 10.57 (N = 4)
Camembert (cheese)	2.76 ± 3.80 (N = 4)
Mozzarella (cheese)	2.94 ± 4.86 (N = 4)
Emmental (cheese)	1.40 ± 2.79 (N = 4)
Sheep’s cheese	7.26 ± 4.98 (N = 4)
Feta (cheese)	1.90 ± 1.78 (N = 4)
Cheddar (cheese)	7.39 ± 13.95 (N = 4)
Raclette (cheese)	6.61 ± 11.97 (N = 4)

RT-PCR inhibition values of <0% have been rounded up to 0%. N: number of samples tested.

## Data Availability

No new data were created or analyzed in this study. Data sharing is not applicable to this article.
